# Miscarriage Prevented

**Published:** 1899-03

**Authors:** J. E. Huffman

**Affiliations:** Healdsburg, Cal.


### MISCARRIAGE PREVENTED.

J. E. Huffman, Healdsburg, Cal.

February 5th, 1897, Mrs. P., Chicago, suffered a miscarriage, and later had one premature birth. She also had one child that lived two years and then died of spinal meningitis, Pregnant at the present date, and has the following symptoms: Cramps in calves at night; has to get out of bed and stand, constipation, stools are large, hard, and dark; sore pain over left eye; feet swell. ℞ Nux-vom., 2c.

February 28th, 1897, was much improved, and I gave, as a constitutional remedy, Calc-c. 45m.

April 21st, 1897, delivered a male child; easy labor and good recovery.

This case occurred in my Senior year.

